# Direct current electrotherapy for internal haemorrhoids: experience in a tertiary health institution

**DOI:** 10.11604/pamj.2014.18.145.3119

**Published:** 2014-06-17

**Authors:** Samuel Olatoke, Moses Adeoti, Olayide Agodirin, Abdulwahab Ajape, John Agbola

**Affiliations:** 1 University of Ilorin teaching hospital, Ilorin, Kwara state, Nigeria; 2 Ladoke Akintola University of Technology, Ogbomoso, Oyo state, Nigeria

**Keywords:** Haemorrhoids, electrotherapy procedure, generator, successful treatment

## Abstract

**Introduction:**

Haemorrhoids disease is one of the most frequently occurring disabling conditions of the anorectum. We re-present the method, advantages and results of using direct current electrotherapy in the treatment of haemorrhoids.

**Methods:**

Symptomatic grades 1, 2 or 3 internal and mixed haemorroids were treated. Exposure and evaluation was with an operative proctoscope which visualized one-eighth of the anal canal at a time. All diseased segments were treated per visit, indicators of successful treatment were, darkening of the treated segment, immediate shrinking of the haemorrhoid and ceasation of popping sound of gas release at the probe tip. Patients were followed up for two weeks. No bowel preparations, medications, anesthesia nor admission was required.

**Results:**

Four hundred and fifty six segments were exposed, 252(55.3%) were diseased. eight patients with either grades 2 or 3 diseases required two treatment visits. The most common symptom was rectal bleeding (94.7%), followed by prolapsed but manually reduced hemorrhoids (68%). Prolapse of tuft of haemorrhoidal tissue with spontaneous return was seen in 59.6%, anal pain in 29.8%, and itching in 3.5%. the median number treated segments per patient was 4. No complication was encountered. All patients treated remained symptom free at a mean duration of follow up of 16 months.

**Conclusion:**

Direct current electrotherapy is an effective, painless and safe out-patient treatment method for grades 1 to 3 internal and mixed hemorrhoid disease.

## Introduction

Haemorrhoid disease, a common human affliction from the dawn of history [1, 2]. It afflicts 50% of adults above 50years of age [3], The exact incidence is however difficult to ascertain because patients often postpone presentation due to personal, socioeconomic and cultural reasons and due to fear of pain, protracted recovery, the cost of open operative treatment [1, 3]. Patients also postpone hospital visitation because of fear of anesthesia and operation and the erroneous believe that operations on haemorrhoids may lead to loss of libido and erectile dysfunction.

Haemorrhoids are enlarged symptomatic anorectal vascular cushions. The cushions are conglomerates of blood vessels, supporting tissues and overlying mucus membrane or skin of the anorectal region. Hemorrhoids can be classified based on the location of the base in relation to the dentate line. If the base is above it is classified as internal hemorrhoids, if the base is below the dentate line it is classified as external [1.4] and if its base spans across the dentate line it is classified as mixed. The Goligher system further classifies internal hemorrhoids into four grades or degrees of prolapse; grade 1 is haemorrhoid not prolapsing outside the anal canal; Grade 2 Prolapses upon bearing down at defecation but retracts spontaneously, grade 3 prolapses upon bearing down at defecation but requires manual reduction and grade 4 is non-reducible (persistently) prolapsed haemorrhoids [1, 4].

Several treatment alternatives have been developed [3–5] in order to eliminate some of the concerns of the treatment modalties, particularly the open hemorrhoidectomy. One of such alternatives is direct current electrotherapy. The direct current electrotherapy was first used in 1867. A comprehensive review of the method was published by Wilbur E.F. Keesey, M.D in 1934 [6]. However, since its introduction this non-operative method of treatment of hemorrhoid has failed to enjoy wide attention in the medical community.

**Figure 1:**
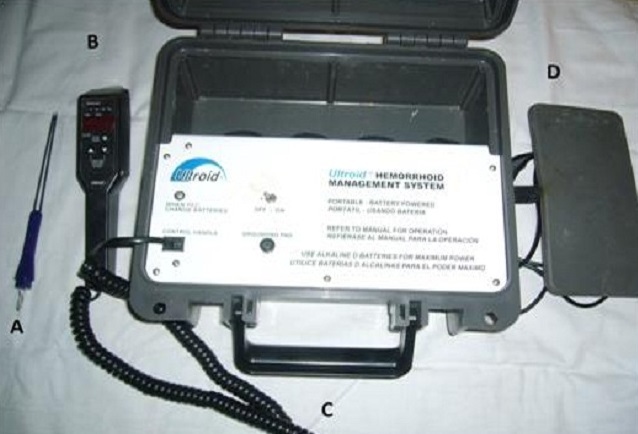
The electrotherapy generator components: A-disposable dual probe tip, B-probe handle, C- the electrotherapy generator, D- grounding pad

The electrotherapy generator (Figure 1 - C) generates dc current from a 220volt alternating current external source. The delivery of dc current to the hemorrhoid is by a probe handle and disposable dual probe tip (Figure 1 - A and B). The handle incorporates milli-ampere and time display controls and also the controls for the timer. The milli-ampere control is for the initiation and cessation of current flow to the probe tip and it is also for up or down regulation of the current. The probe acts as the negative electrode while the grounding pad (Figure 1 - D) is the positive electrode

We re-present in details, the procedure of direct current electrotherapy and a prelimnary report of prospective evaluation of patients who had dc electrotherapy for their internal haemorrhoids in Ilorin, north central Nigeria.

## Methods

After obtaining ethical clearance from the hospital, patients who presented with hemorrhoid disease during the 18 months study period between July 2009 and January 2011 were offered an option of open operative treatment or dc electrotherapy. All patients with stages 1 to 3 hemorrhoids who opted for the latter therapy and gave informed consent were included in the study. Their histories were reviewed, digital rectal and proctoscopic examination were done. Patients with causes other than haemorrhoid disease accounting for their symptoms were excluded. Those with grade 4 haemorrhoids were also excluded because of difficulty with manual reduction of the haemorrhoids and because insertion of the proctoscope provokes pain which hampers proper assessment, especially when there is ulceration. Patients? information was collected in pre- designed proforma. We did not compare the result with those of the preexisting treatment method of open hemorrhoidectomy.

No medication, including prophylactic antibiotics, was given before the procedure neither was bowel preparation done. Proctoscopy was performed using the Hinkle - James rectal speculum with an operative port which exposes a segment equal to one - eighth of the circumference of the anorectum at a time. Non-anesthetic, KY, gel was used to lubricate.

With the patient in Sims position the grounding pad is positioned securely under the left thigh. For the hemorrhoids on the left and right sides, the probe tip assembly is secured in the treatment handle in the horizontal position while for the anterior and posterior hemorrhoids the probe tip assembly is secured in vertical position. Using the proctoscope, eight segments were exposed and assessed in each patient, starting from the right side of the patient and working clockwisely.

The target hemorrhoid is isolated in the proctoscope's operative port. The probe tip is positioned on the base of the haemorrhoid above the dentate line at a slight angle to the anal canal. If the patient perceives the touch of the probe tip then it is repositioned until the sensation is lost before initiating the current flow. After initiation, the current is raised to 2MA. While the current flows the probe tip is advanced about 0.5cm into the haemorrhoidal vessels. Further penetration is prevented by a covering insulator on the probe tip. The current is increased at intervals of 1 to 2 minutes to a maximum of 16 MA or to patient’s tolerance below 16MA. When the treatment of the segment is completed, the current is slowly depressed to zero. All diseased haemorrhoid segments are treated per office visit, starting with the highest grade lesion.

Indicators of successful treatment were darkening of the treated segment; this suggests thrombosis. Cessation of the popping sounds of gas release at the probe tip indicating cessation of blood flow at the treated position and immediate shrinking of the hemorrhoid. Where residual hemorrhoids are present after the initial treatment, the patient returns for repeat treatment after 4 weeks. Follow-up was by phone contact. If symptoms suggestive of haemorrhoid disease were elicited, the patient was invited for evaluation. Measure of outcome was resolution of all symptoms, patients that have been considered healed did not have further proctoscopy as long as they remained asymptomatic on follow-up for two weeks. Statistical analysis of data was done using SPSS 16.( SPSS Inc)

## Results

Fifty Seven patients, 49 males, and 8 females, were included in this study, the mean age was 42 years (range 18-60)

The median duration of symptoms was 24 months (range 4-216) months. Patients with higher grade disease appeared to have harbored the disease longer than patients with lower grade disease but there was no significant difference in median duration of disease per grade of disease (Table 1) P = 0.22

**Table 1:** Duration of disease

Duration/months	Grade 1	Grade 2	Grade 3
**4**	-	2	-
**6**	-	3	-
**10**	1	2	3
**12**	-	3	1
**20**	-	-	1
**23**	-	1	-
**24**	2	9	4
**36**	1	3	-
**48**	-	3	2
**60**	-	1	-
**72**	-	3	5
**96**	-	-	1
**120**	-	2	4
**144**	-	1	1
**216**	-	-	1
**P = 0.22**			

Two patients had treatment with dc-electrotherapy for recurrent haemorrhoids after surgical haemorrhoidectomy while 34 patients had tried only medical therapy including topical creams, suppository and stool bulking agents before electrotherapy

The most common symptom was rectal bleeding (94.7%), 68% presented with grade 2 prolapse, 59.6% with grade 1 prolapse. The presentation included anal pain in 29.8%, and itching in 3.5% of the patients.

Three patients had Grade 1 disease, 32 patients had grade 2, while 22 patients had grade 3 disease. Eight segments were exposed and evaluated per patient giving a total of 456 exposed segments, 252 (55.3%) of these segments were treated for haemorrhoidal disease. The median number of segments of haemorrhoid disease per patient was 4. There were 13, 147, and 92 segments with grades 1, 2 and 3 diseases respectively

Most patient, 86% (49), were successfully treated in one visitation, 8 patients had symptomatic residual grade 1 diseases requiring more than one office session. Two of these 8 patients presented initially with grade 2 disease while the remaining had grade 3 diseases. No patient experienced any side effect during the treatment and follow up period. Most of the patients tolerated 10 -16 MA of current for a period of 10-12 minutes per segment

## Discussion

The treatment of hemorrhoid disease may be accompanied by varying degrees of operative difficulties, varying length of hospital stay and varying severities of postoperative morbidities including pain, acute retention of urine, peri-anal sepsis and discharge, postoperative hemorrhage, recurrence, anal stenosis and allergic reaction [2, 3, 7] depending on the modality employed. A modality of treatment with an instrument which incorporates safety and ease of operation provides an effective, safe and painless procedure for outpatient treatment of internal and mixed haemorrhoid disease is the subject of this article.

Direct current electrotherapy was applied to 250 haemorrhoid segments in 57 patients. The median number of haemorrhoid segments per patient was 4. The procedure was done as an office procedure without any anesthesia.

In 49 patients (86%), symptoms resolved after one treatment session. A second session was required in 8 patients (18%). All patients requiring a second treatment had either grade 2 or grade 3 diseases. No treatment failures were encountered. This may be as a result of patient selection whereby all grade 4 patients were excluded. In a study by Aziz et al [2] with similar number of patients, the complete response rate was 82%, the partial response rate was 10% and treatment failure rate was 8%. When compared with our findings the treatment outcomes were not different (P = 0.61).

In our observation, pain was not a complication contrary to documented by Aziz et al [2]. But some patients had dull non-localized rectal aching sensation during the procedure which resolved upon depression of the current. Aside from this aching sensation and the recurrence of lesser grade diseases in some patients as earlier noted, no other complications during the procedure or follow up period. This attests to the safety of the procedure if appropriately used. Direct current in milliampere and time of application was directly related with disease grade[8]

This procedure was possible as an office procedure for two reasons. First, absence of somatic sensory innervations above the dentate line[9]. is responsible for the absence of pain.. Secondly, after the initiation, the current flow was raised slowly. The slow up-regulation of the current flow permits tolerance. The precise mechanism of action of D.C. electrotherapy that leads to resolution of the disease is not known [8]. Thrombosis of the haemorrhoidal vessels with subsequent resorption or sloughing is postulated [10]. The vascular thrombosis is believed to be due to several mechanisms; Generation of heat at the probe tip, Direct trauma to the haemorrhoid vascular network by the probe tip, and spasm of the haemorrhoidal vessels or their vasa vasorum [2, 11, 12].

During the procedure, break down or sodium chlorine and hydrolysis of water in the tissue leads to production of sodium hydroxide salt (NaOH) and the release of free chlorine gas. It is suggested that the NaOH causes tissue destruction resulting in the release of thrombogenic substances. Furthermore, the free chlorine gas may cause direct tissue damage and when oxidized may contribute to the thrombogenesis. This chemical reactions results in loss of tissue volume which explains the observed tissue shrinkage [6].

## Conclusion

Direct current electrotherapy appears to offset many concerns raised with the use of other methods in particular the open hemorrhoidectomy. However, further studies including randomized controlled trial using equal grades of disease will be required for the appropriate placement of this treatment modality and to determine its acceptance. Some observed benefits of dc electrotherapy are; no requirement for anesthesia, no premedication, no bowel preparation, and no admission hence the patients can resume their normal activities immediately. A set back of this modality of treatment of hemorrhoid is that more than one treatment session may be required for grade 3 diseases and the procedure may not be applicable to grade 4 diseases.
